# Isomers and energy landscapes of micro-hydrated sulfite and chlorate clusters

**DOI:** 10.1098/rsta.2017.0154

**Published:** 2018-02-05

**Authors:** John C. Hey, Emily J. Doyle, Yuting Chen, Roy L. Johnston

**Affiliations:** School of Chemistry, University of Birmingham, Birmingham B15 2TT, UK

**Keywords:** sulfite, chlorate, water, gas-phase, hydration, nucleation

## Abstract

We present putative global minima for the micro-hydrated sulfite SO_3_^2−^(H_2_O)_*N*_ and chlorate ClO_3_^−^(H_2_O)_*N*_ systems in the range 3≤*N*≤15 found using basin-hopping global structure optimization with an empirical potential. We present a structural analysis of the hydration of a large number of minimized structures for hydrated sulfite and chlorate clusters in the range 3≤*N*≤50. We show that sulfite is a significantly stronger net acceptor of hydrogen bonding within water clusters than chlorate, completely suppressing the appearance of hydroxyl groups pointing out from the cluster surface (dangling OH bonds), in low-energy clusters. We also present a qualitative analysis of a highly explored energy landscape in the region of the global minimum of the eight water hydrated sulfite and chlorate systems.

This article is part of the theme issue ‘Modern theoretical chemistry’.

## 1. Introduction

Ions can be classified according to the Hofmeister series, which orders ions according to their ability to de-solvate proteins [1–5]. The ions which promote disorder within the hydrogen-bonding network of water and disrupt protein stability are labelled as chaotropes, while ions which promote long-range order and protein stability within water are classified as kosmotropes [1,2,6]. The hydration of ions is also important as it has a large bearing on the atmospheric nucleation of water clusters and other areas of atmospheric science [7–9]. We have previously studied the chaotropic perchlorate (ClO_4_^−^) and the kosmotropic sulfate (SO_4_^2−^) ions in finite gas-phase water clusters [10–12].

The SO_2_ released from natural and synthetic sources is quickly oxidized in the atmosphere to form sulfur(IV) and sulfur(VI) species. Sulfite (SO_3_^2−^) and other sulfur(IV) ions are commonly found in inorganic aerosols, which are important in atmospheric science as they contribute to the formation of acid rain [8,13–15]. There have been several studies focusing on sulfite in the past [8,16,17] and sulfite has been shown to be kosmotropic in molecular dynamics simulations with model proteins [17]. Chlorate (ClO_3_^−^) is isoelectronic with sulfite and shares a similar geometry, with a lower charge and thus provides a useful comparison with sulfite.

## 2. Methods

An empirical potential has been chosen to allow for fast and efficient searching of a large range of cluster sizes. The interaction energy (*U*) is evaluated as the sum of Coulombic and Lennard–Jones contributions over all pairs of interacting sites (atoms and pseudoatoms);
2.1
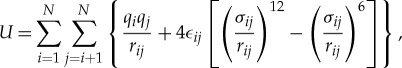
where *r*_*ij*_ is the inter-site separation, *σ*_*ij*_ and *ϵ*_*ij*_ are the Lennard–Jones energy and distance parameters, and *q*_*i*_ and *q*_*j*_ are the charges on sites *i* and *j*, respectively.

The Lennard–Jones parameters used for sulfite were derived using Moller–Plesset MP4(SDTQ) *ab initio* calculations [18]. The sulfite ion is modelled as a rigid pyramidal species with O–S–O bond angles of 106^°^ and S–O bond lengths of 1.9 Å. The sulfur atom is 0.76 Å out of the plane of the three oxygen atoms. The Lennard–Jones parameters and partial charges used for chlorate were taken from the literature and were determined by *ab initio* LCAO MO SCF calculations [19,20]. The chlorate ion is also modelled as a rigid pyramidal species, the O–Cl–O bond angles are 105.8^°^ and the Cl−O bond lengths are 1.48 Å. The chlorine atom is 0.58 Å out of the plane of the oxygen atoms. The partial charges for both ions were calculated in this work using the Bader method [21] within the NWChem [22] DFT package using the B3LYP exchange correlation functional and the 6-311++G** basis set, the charges were calculated for a lone ion in the gas phase.

Water molecules in this study are modelled using the TIP4P potential: a rigid four-site molecule is defined with a H−O−H bond angle of 104.52^°^, with an oxygen lone-pair site represented by a pseudoatom which carries the charge of the oxygen atom but has no Lennard–Jones parameters. The TIP4P potential has been shown to replicate many of the properties of bulk water, while also being computationally inexpensive to model [23–25]. The Lennard–Jones and partial charges are listed in table 1.

**Table 1.** Table of Lennard–Jones parameters and partial charges used for sulfite, chlorate and TIP4P water in this study [18].

site	*ϵ* (kcal mol^−1^)	*σ* (Å)	*q*(*e*)
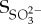	0.25	3.6	0.281
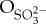	0.25	3.2	−0.760
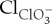	0.040	4.86	0.452
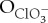	0.076	3.10	−0.484
O_H_2_O_	0.648	3.2	0
H_H_2_O_	0	0	0.52
LP_H_2_O_	0	0	−1.04

Low-energy minima on the potential energy surface for the ion-water clusters were explored using the basin-hopping Monte Carlo algorithm developed by Wales and co-workers within the pele package [26,27]. Basin-hopping was used to identify a putative lowest energy structure (global minimum, GM), to efficiently search the potential energy landscape and to create a database of low-energy minima for later mapping of the landscape [26,28]. Ten basin-hopping runs of 500 000 steps were performed in parallel for each size of 
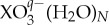
 cluster in the range 3≤*N*≤50.

The geometry perturbations implemented in this study were performed in blocks of 100 moves of the same type, with minimizations performed after each perturbation. The three move classes implemented are random translations, random rotations and cycle inversion moves. The translation moves apply random translation vectors to molecules within the cluster. Rotation moves apply random rotation vectors in the range ±*π* radians to all of the molecules within the cluster. The cycle inversion moves were performed using the method outlined in our previous work [10–12], first proposed by Takeuchi [29].

For the landscape exploration of the hydrated sulfite and chlorate systems, the doubly nudged elastic band method [28] was used to search for transition states between minima found during global optimization. Pathways are found by sampling between end points using a linear interpolation of the translational coordinates of the rigid-body molecules and spherical linear quaternion interpolation of the rotational coordinates [30]. Candidate transition states are then further optimized using hybrid eigenvector following [31,32]. The resultant energy landscapes are visualized as disconnectivity graphs using the PyConnect package [33].

## 3. Results

### (a) Global optimization

**Figure 1.**
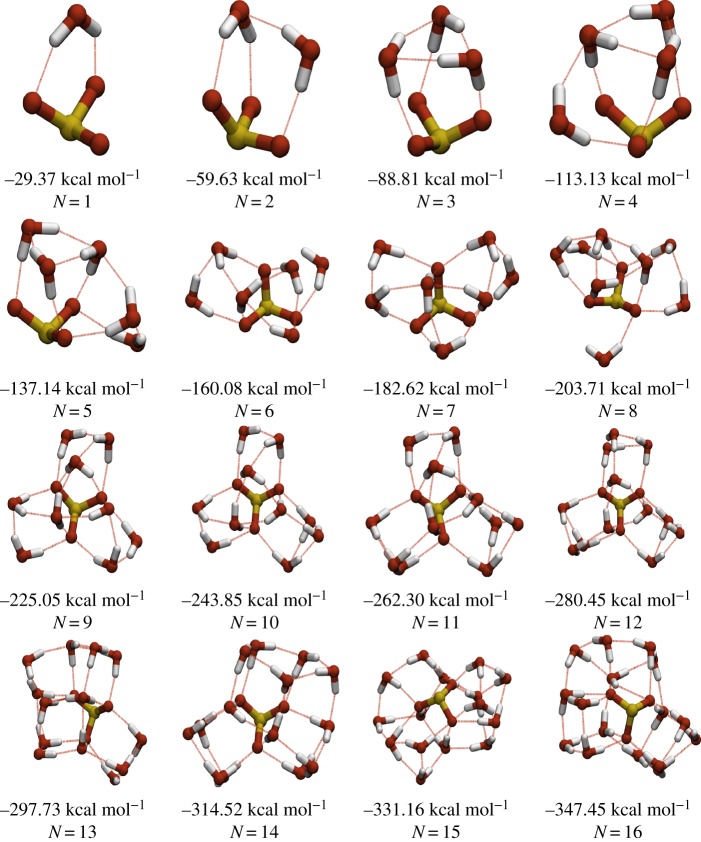
Table of lowest energy minima for the SO_3_^2−^(H_2_O)_*N*_ system in the range 3≤*N*≤16. Electronic supplementary material, ESM2, contains the coordinates and energies for the putative global minima in the range 3≤*N*≤50. (Online version in colour.)

The putative GM structures for SO_3_^2−^(H_2_O)_*N*_ clusters in the range 1≤*N*≤16, which are shown in figure 1, display a preference for high-symmetry structures at small sizes.

**Figure 2.**
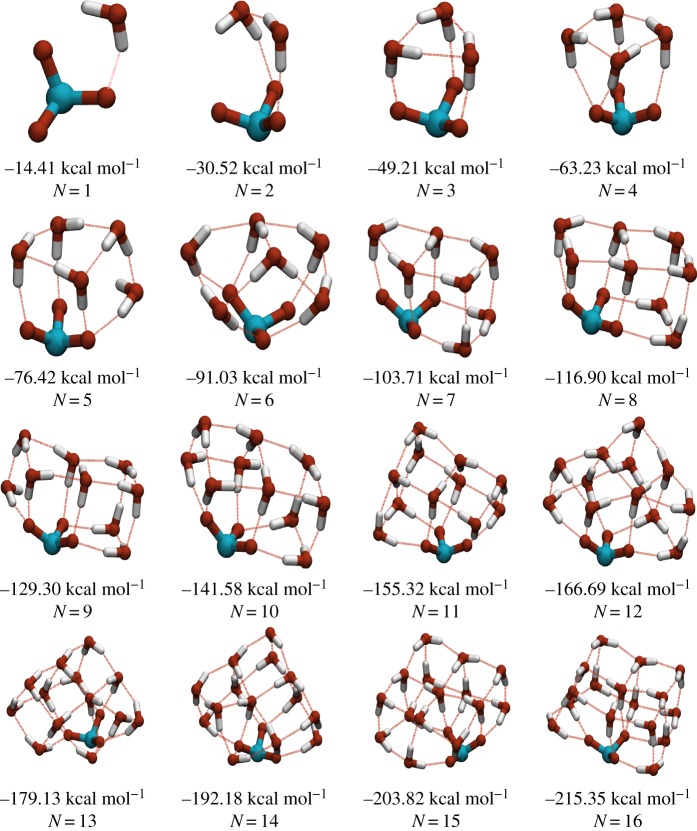
Table of lowest energy minima for the ClO_3_^−^(H_2_O)_*N*_ system in the range 3≤*N*≤16. Electronic supplementary material, ESM1, contains the coordinates and energies for the putative global minima in the range 3≤*N*≤50. (Online version in colour.)

For structures with *N*≥19, the water molecules complete the first solvation shell around the sulfite ion, which adopts a central position in the cluster. There is a void within the cluster inside a cage-like water structure above the sulfur atom of the sulfite for structures with *N*≥19, although there is some coulombic attraction between the positively charged S atom and the negative pseudoatom of the TIP4P water molecules, which carries the partial charge of the oxygen site. The small sulfite clusters (*N*≤12) show a high preference for the formation of trimeric hydrogen bond rings, with all of the clusters in this range exhibiting trimeric rings as the only closed loops in their hydrogen-bonding networks, which is similar to the behaviour observed for sulfate ions in previous work [11]. The putative global minima for the chlorate system are presented in figure 2. The hydrated chlorate clusters generally have structural motifs similar to those seen in finite water clusters, displaying a preference for stacked cubes, pentagons and hexagons, which are commonly seen in water clusters [34]. The chlorate ion displays a marked preference for occupying surface sites in the clusters, occupying these sites for all sizes of cluster studied. This preference for water-like structural motifs leads to the chlorate clusters generally having lower symmetry than the corresponding sulfite clusters. As the size of the clusters increases, both systems exhibit larger hydrogen-bonding rings, which are common in large pure-water clusters. At these larger sizes, the sulfite continues to occupy sub-surface sites, and the chlorate continues to occupy surface sites.

**Figure 3.**
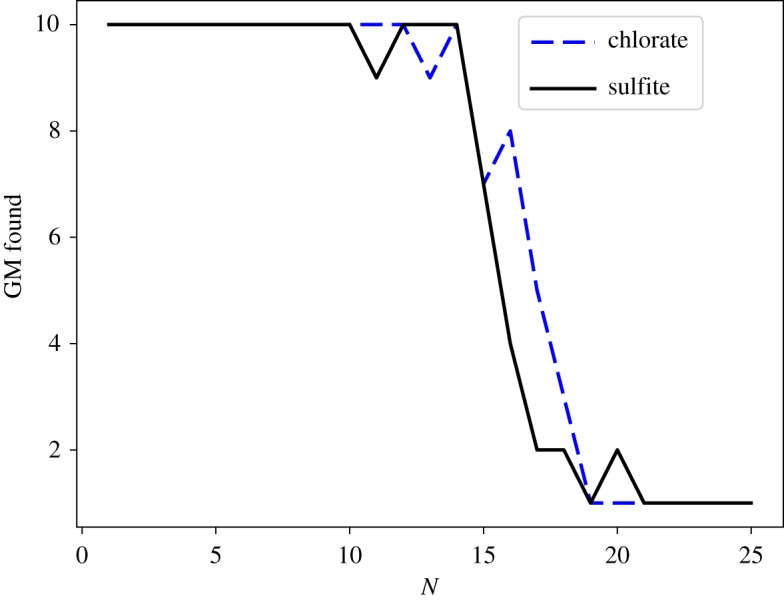
Number of parallel basin-hopping runs which converge on the same lowest energy minimum (GM) structure for hydrated chlorate and sulfite ions with 1–25 water molecules. (Online version in colour.)

From figure 3, it can be seen that in the range 1≤*N*≤15, the same lowest energy structure is found by the majority of the independent basin-hopping runs for both ionic systems. For larger sizes, the number of independent runs converging on the same minimum falls rapidly, reflecting the rapid rise in the number of minima with cluster size. The confidence in having identified the true GM for both systems decreases, but we still believe that we find a representative sample of low-energy minima on which analysis can be performed.

For verification of our potential, we locally re-optimize a number of low-energy minima at the density functional theory (DFT) level, using the B3LYP exchange correlation functional and the 6-311++G** basis set and D3 dispersion corrections, as implemented within the NWChem package [22].

**Figure 4.**
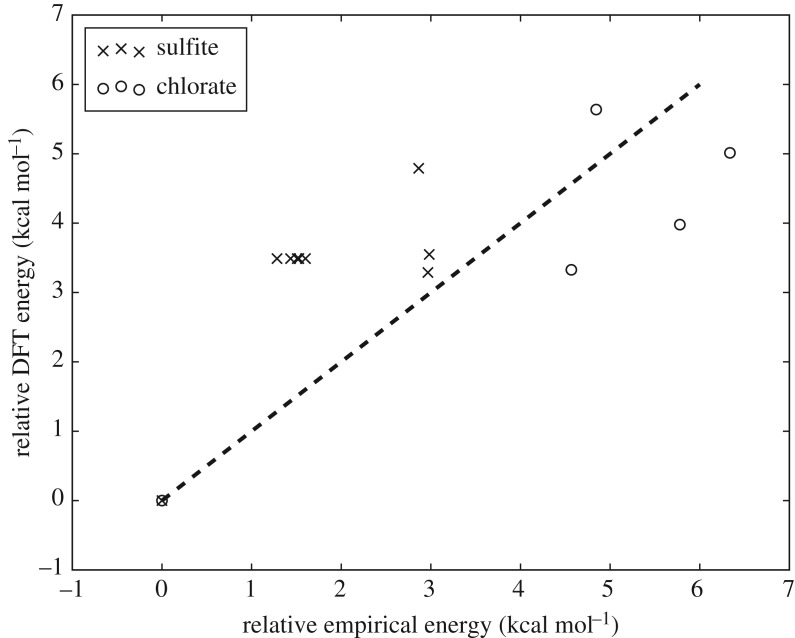
Locally minimized DFT versus empirical energies for the 10 lowest energy minima found for *N*=3 on the empirical landscapes of the sulfite and chlorate systems. Energies are given relative to the respective global minima. The dashed line corresponds to the location of perfectly correlated minima.

Figure 4 shows the correlation between the DFT and empirical energies for the 10 lowest energy SO_3_^2−^(H_2_O)_3_ and ClO_3_^−^(H_2_O)_3_ minima when re-optimized at the DFT level. The lowest energy minimum found at the empirical level remains the lowest in energy at the DFT level for both the chlorate and sulfite systems. Some of the initial minima are almost degenerate in energy, and thus there is some overlap. Several of the non-degenerate sulfite minima re-optimize to the same minimum on the DFT landscape. This simplification of the DFT landscape when compared with the empirical landscape has been noted in some of our previous work [10]. The re-optimized minima all share the same oxygen framework as their parent empirical minima, with some lengthening seen in the water–water hydrogen bonds. This would likely be improved by the use of a polarizable empirical water model. When re-optimizing small numbers of minima for larger sizes (up to *N*=12), there is generally a good agreement between the energetic ordering of the empirical and DFT minima, although this does worsen as expected for larger sizes. The agreement is perhaps not surprising, as the empirical potentials for chlorate and sulfite are fitted to high level methods [18–20] and the charges used for the ions are calculated using the same DFT functional and basis set as used in our re-optimizations [22]. This is consistent with the observations made in our previous work [10–12].

**Figure 5.**
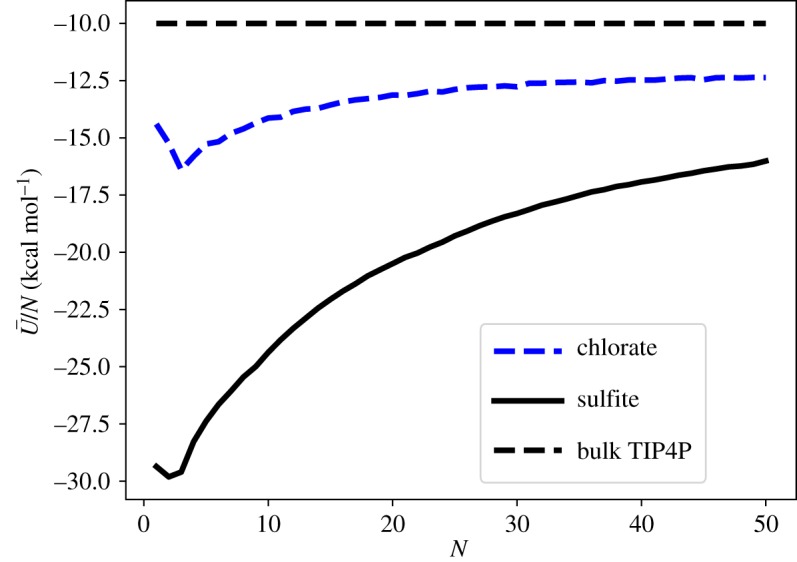
Boltzmann weighted interaction energy per water molecule for SO_3_^2−^(H_2_O)_*N*_ and ClO_3_^−^(H_2_O)_*N*_ clusters in the range 1≤*N*≤50. (Online version in colour.)

The evolution of interaction energy per water molecule in the range 1≤*N*≤50, shown in figure 5, is obtained by Boltzmann weighting according to equation (3.1):
3.1
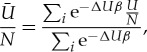
where Δ*U* is the interaction energy of cluster *i* above the putative GM, *β*=1/*k*_B_*T* and the average interaction energy per water molecule is denoted by 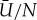. The weighted mean is taken over all structural isomers of unique energy found in all basin-hopping runs for each size of cluster. A theoretical temperature of 130 K is used to maintain consistency with previous computational work [10,11] based on the experimental studies of the hydrated sulfate ion [14]. Figure 5 shows that the asymptotic limit for the interaction energy for TIP4P water in the bulk (≈−10 kcal mol^−1^) has not yet been reached at *N*=50. The decreasing magnitude of 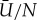 with increasing *N* is due to the dilution effect of the single ion. The deviation from the TIP4P asymptote is greatest for sulfite–water clusters, due to the higher charge of the sulfite ion, leading to a larger coulombic interaction between the TIP4P water molecules and the ion.

It was shown experimentally by Williams and co-workers [14] and computationally by Smeeton *et al.* [11] that the sulfate ion (SO_4_^2−^) promotes long-range order within water nano-droplets, with total suppression of hydroxyl groups protruding from the surface of the cluster, (dangling O−H bonds), for all sizes in the range 18≤*N*≤43.

### (b) Structural characterization

**Figure 6.**
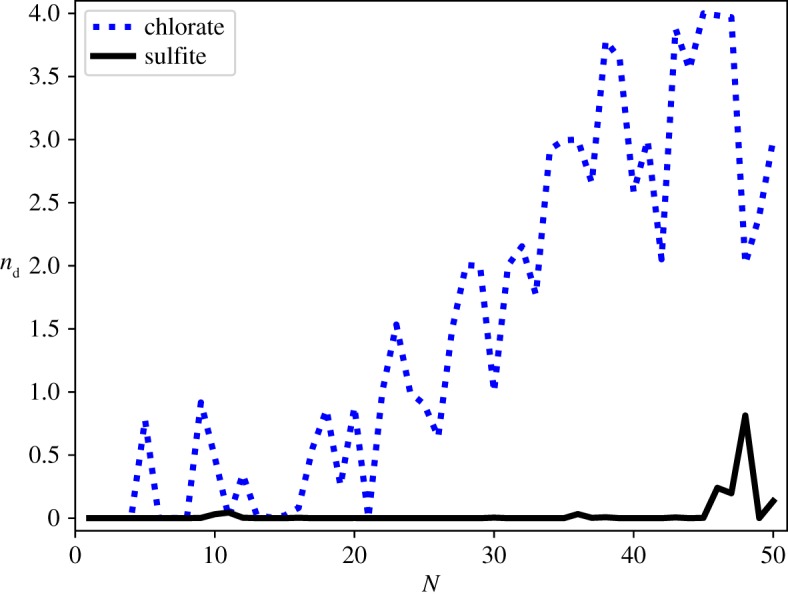
Boltzmann weighted mean number of dangling hydrogen bonds (*n*_d_) for SO_3_^2−^(H_2_O)_*N*_ and ClO_3_^−^(H_2_O)_*N*_ clusters in the range 1≤*N*≤50. (Online version in colour.)

Figure 6 shows that the sulfite ion also promotes long-range order within the water clusters by totally suppressing the appearance of dangling O−H bonds for every optimized cluster found with Δ*U*≤5 kcal mol^−1^ in the range 1≤*N*≤50. The presence of the sulfite ion induces all the hydrogen atoms of the water molecules in the clusters to interact either with the oxygen atoms of the other water molecules or directly with the oxygen atoms of the sulfite ion. Owing to the higher negative charge localized on the oxygen atoms of the sulfite ion (since the negative charge is spread only over three oxygen atoms), this effect is stronger for sulfite than sulfate, with the effect dominating well into the formation of the third solvation shell. This effect is seen, to a lesser degree, in the chlorate system, with the chlorate ion providing a greater degree of patterning than the perchlorate system [10]. There is complete suppression of the appearance of dangling O−H bonds in chlorate–water clusters with 3≤*N*≤18. In the larger clusters, there are fewer unbonded hydroxyl groups than are reported for the equivalent perchlorate clusters, for all sizes.

**Figure 7.**
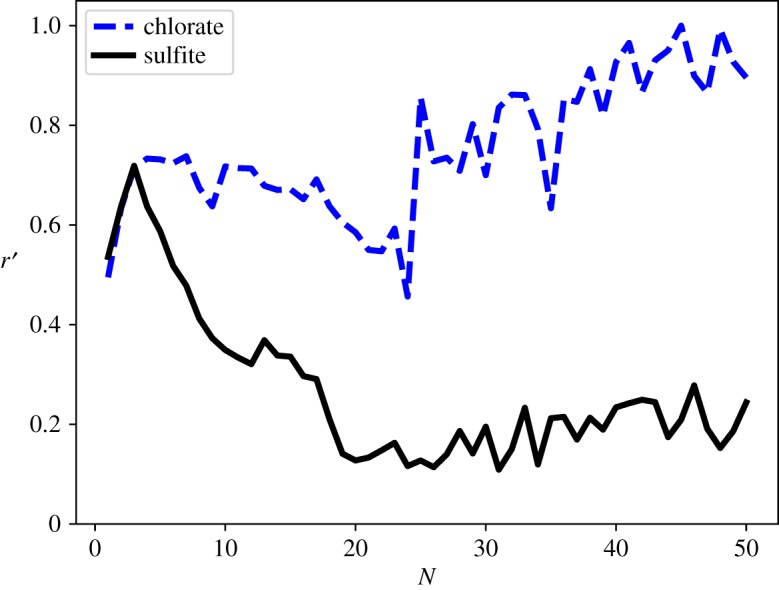
Displacement *r*^′^ of the chlorate and sulfite ions within the cluster in the range 1≤*N*≤50. (Online version in colour.)

The ratio of the displacement, (*r*), of the centre of mass of each ion to the radius of gyration, (*r*_gyr_), of the cluster for the 1000 lowest energy clusters is Boltzmann weighted according to the following equation:
3.2
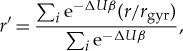
where Δ*U* is the energy of minimum *i* above the GM, *r* is the displacement of the centre of mass of the sulfite ion from the centre of mass of the water molecules in cluster *i*, and *β*=1/*k*_B_*T*. Figure 7 shows that for clusters in the range 1≤*N*≤8 the sulfite ion lies on the surface of the cluster, i.e. the formation of the water cage around the sulfur site has not yet begun. For clusters with 12≤*N*≤19, there is a decreasing displacement of the ion from the centre of the cluster as the cage forms around the sulfur atom, and thus the first solvation shell is completed. The displacement then remains relatively constant until *N*=29, due to the spherical growth of the second hydration shell. At larger sizes, as the third solvation shell forms, we see the emergence of features very common to pure water clusters, namely stacked cubes, pentamers and hexamers, although we do not see the emergence of the dangling hydrogen bonds which are normally prevalent in those structural features in pure water clusters. The sulfite does not remain at the centre of the cluster, but instead favours subsurface sites.

**Figure 8.**
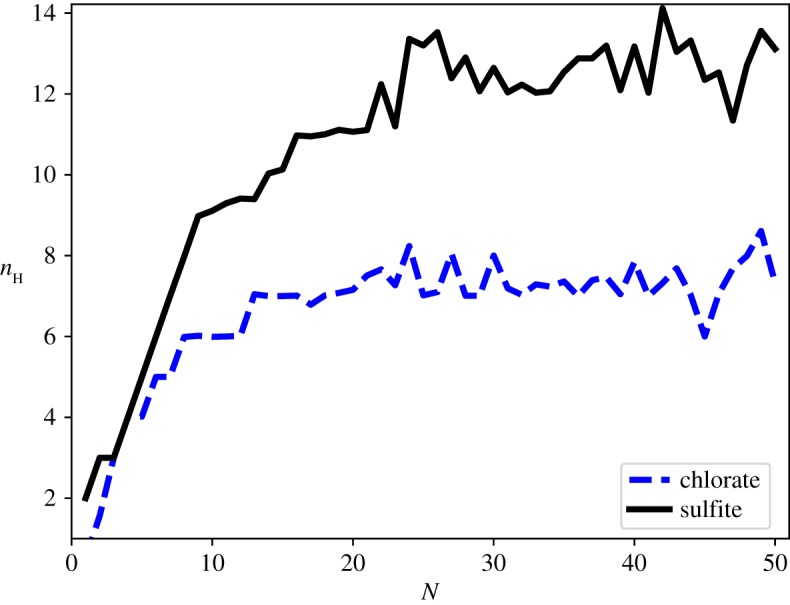
Boltzmann weighted mean number of hydrogen bonds *n*_H_ coordinated to the ion in the range 1≤*N*≤50. (Online version in colour.)

The Boltzmann weighted mean total number of hydrogen bonds donated by the water molecules of the cluster to the ion is shown in figure 8. The Boltzmann weighting is performed across the 1000 lowest energy minima identified by the global optimization. The sulfite ion consistently accepts a greater number of hydrogen bonds than the chlorate ion. This is consistent with the preference of the more highly charged sulfite ion for subsurface sites in contrast with the chlorate which favours surface sites, with the sulfite ion being more accessible to donating water molecules than the chlorate ion at each size. The sulfite ion is observed to coordinate to up to around 14 water molecules with four/five hydrogen bonds donated to each oxygen atom in the ion. The chlorate ion, in contrast, coordinates to a maximum of nine water molecules, with a maximum of three hydrogen bonds donated to each oxygen atom.

### (c) Energy landscapes

The energy landscapes of the SO_3_^2−^(H_2_O)_8_ and ClO_3_^−^(H_2_O)_8_ systems were extensively searched using the method outlined above. The potential energy landscape seen for these cluster sizes is typical of the landscapes searched for the small clusters, and is presented here because they are the most extensively searched.

**Figure 9.**
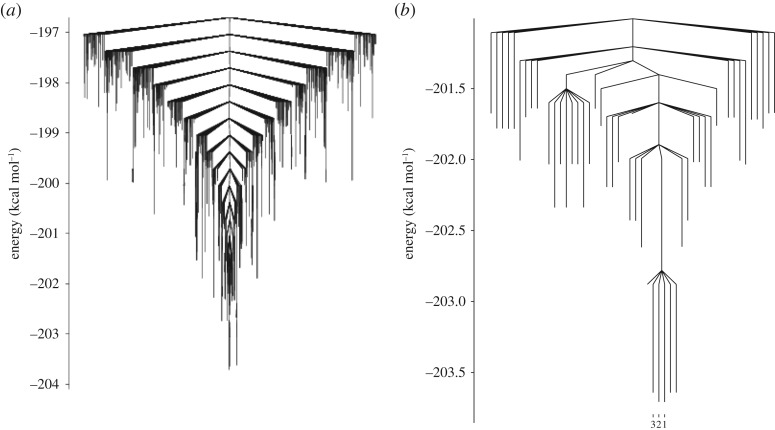
Disconnectivity graphs showing the putative GM and connected minima for the SO_3_^2−^(H_2_O)_8_ system. The explored landscape comprises 18 860 minima and 425 478 connecting transition states. The graphs show connected minima within (*a*) 7 and (*b*) 2.8 kcal mol^−1^ of the GM, with the three lowest energy minima labelled in (*b*).

**Figure 10.**
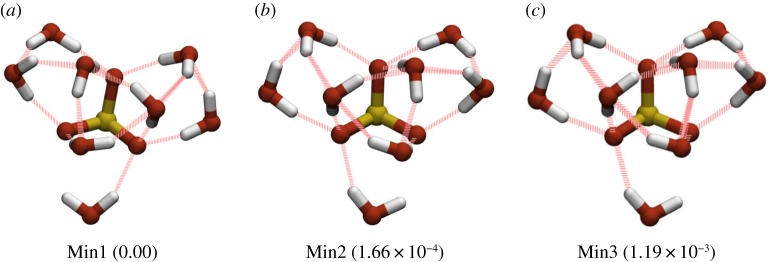
Three lowest energy minima for SO_3_^2−^(H_2_O)_8_. Energies (in kcal mol^−1^) relative to the GM energy are given in parentheses. (Online version in colour.)

It can be seen from figure 9 that there is a strong directing effect across the wider sulfite landscape towards the basin containing the proposed GM. The landscape is, therefore, highly funnelled, with relatively low barriers separating local minima from the GM basin. This, coupled with the relatively low number of minima and the efficiency of the basin-hopping search algorithm, explains why sulfite-water clusters in the size range 3≤*N*≤13 are found in all 10 basin-hopping runs. Figure 10 shows the three lowest energy minima found in the landscape of the SO_3_^2−^(H_2_O)_8_ system. There is a barrier of 0.8 kcal mol^−1^ separating the GM (Min1) from local minimum (Min2). These minima are almost degenerate in terms of energy, they have the same oxygen framework, with the only structural difference being a reversal of the direction of the trimeric hydrogen bond ring. The three next lowest minima are slightly higher in energy, and the barrier from the GM is around 0.8 kcal mol^−1^. One of these minima is pictured in figure 10 (Min3). These minima have similar oxygen frameworks to the GM, but with the trimeric water rings rotated relative to the sulfite oxygens.

**Figure 11.**
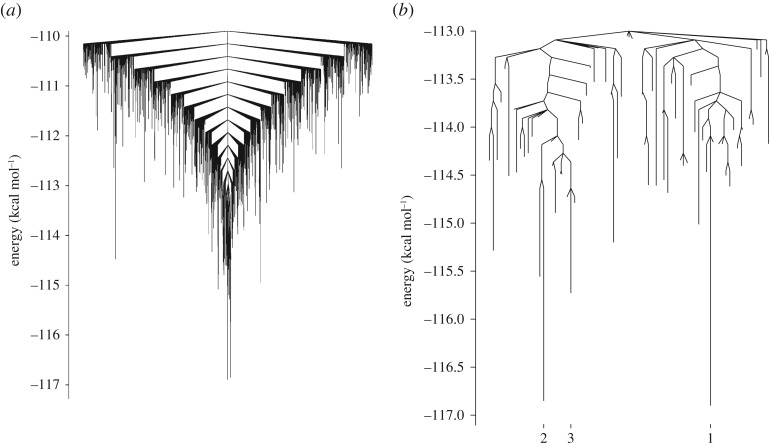
Disconnectivity graph showing the putative GM and connected minima for the ClO_3_^2−^(H_2_O)_8_ system. The explored landscape comprises 36 374 minima and 929 171 connecting transition states. Showing connected minima within (*a*) ≈7 kcal *mol*^−1^ of the GM and (*b*) ≈ 4 kcal mol^−1^ of the GM, with the three lowest energy minima highlighted.

**Figure 12.**
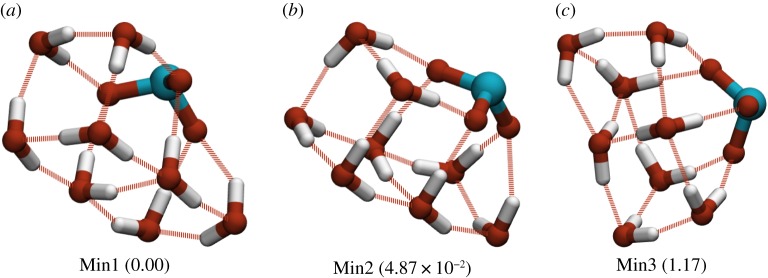
Three lowest energy minima for ClO_3_^−^(H_2_O)_8_. Energies (in kcal mol^−1^) relative to the GM energy are given in parentheses. (Online version in colour.)

The landscape for the ClO_3_^−^(H_2_O)_8_ system is presented as a disconnectivity graph in figure 11. This landscape is well searched with approximately 36 000 minima and approximately 930 000 connected transition states. There is a pronounced funnelling effect also seen in this landscape, but it is less pronounced than that which is seen in the sulfate system. There is a relatively large barrier of around 3.5 kcal mol^−1^ separating the GM from the next lowest energy minimum. This barrier corresponds to an inversion in the direction of a tetrameric hydrogen bond cycle, this can be seen in figure 12. The GM (Min1) and the next lowest energy minimum (Min2) both share the same oxygen framework, which takes the form of a double-stacked cube similar to the GM found for the pure TIP4P_12_ cluster [34]. In these 8-water chlorate clusters, the oxygens of the chlorate take the place of four of the water molecules in the structure. As the chlorate acts as a net acceptor of hydrogen bonds, this leads to the suppression of the four dangling hydrogen bonds normally seen in the equivalent pure water cluster. The next lowest energy minimum (Min3) adopts a different structure to the other two minima, forming a single cube, with two water molecules capping one face.

**Figure 13.**
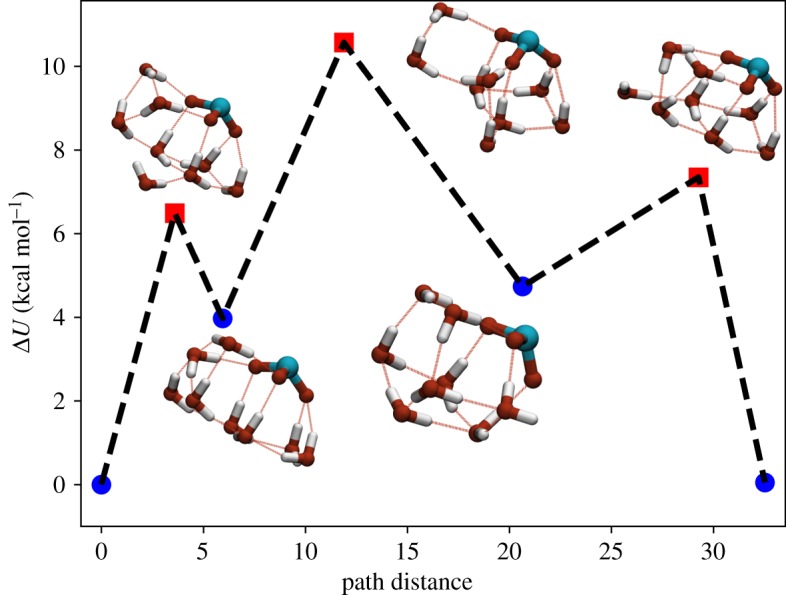
Plot of the pathway between the putative GM (Min1, left) and the second lowest energy minimum (Min2, right) for the ClO_3_^−^(H_2_O)_8_ system. This pathway consists of an inversion in the direction of the hydrogen bond tetramer. (Online version in colour.)

The pathway for the transition between Min1 and Min2 has been extracted and the energies of the minima and transition states are presented in figure 13. The first transition state has an energy of 6.5 kcal mol^−1^ above the GM and corresponds to a significant lengthening of one of the hydrogen bonds in the ring as a water molecule moves out of position. This allows the other hydrogen bonds in the ring to relax and form a trimer. The next-transition state corresponds to another hydrogen bond breaking, to give just two water dimers. This can then relax into a stacked pentamer arrangement with the chlorate oxygens occupying two of the water sites, in a structure similar to the GM of the pure TIP4P_10_ cluster. The next-transition state corresponds to the reformation of a trimeric water ring, before this relaxes to reform the water tetramer.

## 4. Conclusion

We present putative global minima for the hydrated sulfite and chlorate systems in the range 3≤*N*≤16. Using our simple potential model, the sulfite ion is shown to prefer sub-surface sites to surface sites within the water cluster. We show that the sulfite ion exhibits a greater patterning effect on the hydrogen-bonding networks of clusters than the strong kosmotropic sulfate ion. Conversely, the chlorate ion is shown to favour surface sites on the water cluster, and confers a lesser degree of patterning to the hydrogen-bonding network within the water clusters, the effect is stronger than that previously shown for the perchlorate ion using the same model, but less pronounced than the effect of sulfate.

Extensively searched energy landscapes are presented for the SO_3_^2−^(H_2_O)_8_ and ClO_3_^−^(H_2_O)_8_ systems and presented alongside some of the low-energy minima from the landscapes. There is shown to be a strong funnelling effect towards the formation of the GM in the potential energy landscapes of small sulfite-water clusters. A less pronounced funnelling effect is seen for the chlorate system. In both cases, the funnelling effect is stronger than the equivalent XO_4_^*q*−^(H_2_O)_*N*_ systems.

The obvious future direction of this work is to use a more complex model which accounts for polarization of the ions and water molecules. There are several ways in which we can accomplish this, either by using one of the several polarizable empirical potentials such as iAMOEBA [35,36] or by searching directly at a higher level of theory such as HF or DFT with a small basis set.

## Supplementary Material

Putative global minima for hydrated chlorate ions with 1-20 water molecules.

## Supplementary Material

Putative global minima for hydrated sulfite ions with 1-20 water molecules.

## Supplementary Material

Low energy minima in the chlorate + 8H2O landscape

## Supplementary Material

Low energy minima in the sulfite + 8H2O landscape

## Supplementary Material

Structures for the pathway pictured in Figure 12.

## Data accessibility

Coordinates and energies for structures shown in this work are available as electronic supplementary information in XMol XYZ format.
